# Obesity Measures as Predictors of Type 2 Diabetes and Cardiovascular Diseases among the Jordanian Population: A Cross-Sectional Study

**DOI:** 10.3390/ijerph182212187

**Published:** 2021-11-20

**Authors:** Hana Alkhalidy, Aliaa Orabi, Khadeejah Alnaser, Islam Al-Shami, Tamara Alzboun, Mohammad D. Obeidat, Dongmin Liu

**Affiliations:** 1 Department of Nutrition and Food Technology, Faculty of Agriculture, Jordan University of Science and Technology, Irbid 22110, Jordan; ajorabe15@agr.just.edu.jo (A.O.); kaalnaser17@agr.just.edu.jo (K.A.); tamarazboun@yahoo.com (T.A.); 2 Department of Clinical Nutrition and Dietetics, Faculty of Applied Medical Sciences, The Hashemite University, Zarqa 13133, Jordan; islamk@hu.edu.jo; 3 Department of Animal Production, Faculty of Agriculture, Jordan University of Science and Technology, Irbid 22110, Jordan; mdobeidat@just.edu.jo; 4 Department of Human Nutrition, Foods and Exercise, College of Agriculture and Life Sciences, Virginia Tech, Blacksburg, VA 24061, USA; doliu@vt.edu

**Keywords:** obesity, waist circumference, body mass index, type 2 diabetes, cardiovascular diseases, risk, Jordan

## Abstract

Obesity is strongly associated with cardiovascular diseases (CVD) and type 2 diabetes (T2D). This study aimed to use obesity measures, body mass index (BMI) and waist circumference (WC) to predict the CVD and T2D risk and to determine the best predictor of these diseases among Jordanian adults. A cross-sectional study was conducted at the governmental and military hospitals across Jordan. The study participants were healthy or previously diagnosed with CVD or T2D. The continuous variables were compared using ANOVA, and the categorical variables were compared using the X2 test. The multivariate logistic regression was used to predict CVD and T2D risk through their association with BMI and WC. The final sample consisted of 6000 Jordanian adults with a mean age of 41.5 ± 14.7 years, 73.6% females. The BMI (OR = 1.7, CI: 1.30–2.30, *p* < 0.001) was associated with a higher risk of T2D compared to WC (OR = 1.3, CI: 1.04–1.52, *p* = 0.016). However, our results showed that BMI was not associated with CVD risk, while the WC was significantly and positively associated with CVD risk (OR = 1.9, CI: 1.47–2.47, *p* < 0.001). In conclusion, an elevated BMI predicts a higher risk of T2D, while WC is more efficient in predicting CVD risk. Our results can be used to construct a population-specific intervention to reduce the risk of CVD and T2D among adults in Jordan and other countries with similar backgrounds.

## 1. Introduction

Obesity, an excessive or abnormal fat accumulation, is a major risk factor for several chronic diseases, such as cardiovascular diseases (CVD) and type 2 diabetes (T2D) [1]. In some developed countries, such as the U.S., the prevalence of obesity was 42.4% in adults in 2017–2018, according to the National Health and Nutrition Examination Survey [2]. In the European region, the prevalence of obesity ranged from 14.2% to 32.1% (an average of 23.3%) among adults in 2016 [3]. Compared to developed countries, the developing countries are challenged by the double burden of undernutrition and overnutrition, with limited resources to handle chronic diseases [4]. The prevalence of obesity among adults in developing countries varied according to the country’s income level. For example, in the Middle East and North Africa (MENA) region, the lower-middle-income countries had an average prevalence of 20.5%, while a higher prevalence was reported in upper-middle (25.4%) and high-income (33.1%) countries in 2008 [5]. In Jordan, an upper-middle-income country, the prevalence of obesity was 28.1% in 2016 (21.0% of males and 35.6% of females) [6].

Obesity is associated with a low quality of life and an increased risk of chronic diseases and health outcomes [7]. For instance, obesity, especially abdominal obesity, might be associated with T2D by developing insulin resistance [8]. Obesity might also be related to several cardiometabolic risk factors, such as increased blood pressure, dyslipidemia, inflammation, and endothelial dysfunction [9]. Collectively, insulin resistance and other cardiometabolic risk factors might be associated with an increased risk of CVD among obese individuals [8,9].

Obesity could be determined using the body mass index (BMI) [1], and, more precisely, abdominal obesity could be evaluated by measuring the waist circumference (WC) [10]. BMI and WC were used as risk factors in several T2D risk prediction models [11]. A review of five cohort studies showed that the association of T2D with BMI or WC differed depending on the ethnic groups. So far, several cross-sectional studies showed the WC as a better predictor for T2D [12]. For CVD prediction models, QRISK included BMI in the model as a risk factor for CVD [13], while other models such as Framingham, Reynolds, and the World Health Organization/International Society of Hypertension (WHO/ISH) prediction charts neither incorporated BMI nor WC in the prediction model [14]. An analysis of 58 prospective cohort studies from 17 developed countries showed that obesity and central obesity measures did not improve CVD risk prediction among adults [15]. However, WHO indicated that CVD risk would be higher than the predicted risk in the presence of obesity and central obesity [16].

Referring to their strong association and their role in increasing CVD risk [17,18] and T2D [19,20], identifying the risk of CVD and T2D through obesity and central obesity measurements might be necessary to prevent these diseases. As diseases are caused by a complex interplay between several risk factors, an effective health intervention depends on targeting each risk factor distinctly [21]. Studies showed that a BMI and WC reduction was associated with reducing the risk of developing T2D [22,23] and CVD risk factors [24,25].

As different populations showed differences in the association between BMI/WC and T2D/CVD risk, it is necessary to investigate these associations among adults in Jordan, a developing upper-middle-income country. We also aimed to identify the association between Jordanians’ demographic characteristics and obesity (measured by BMI), abdominal obesity (measured by WC), T2D, and CVD. The results of this study might help in designing future interventions aiming to reduce the prevalence of T2D and CVD among Jordanian adults.

## 2. Materials and Methods

### 2.1. Study Design and Participants

This is a national study carried out using a cross-sectional design, conducted between May 2018 and September 2019. The study was performed at the governmental and military hospitals in Jordan. The sample size was determined based on a total Jordanian population of 9 million, in 2017 [26], a previously reported T2D prevalence of 13.1% [6], and a CVD mortality rate of 37% [27] among Jordanians in 2016. The minimum number of samples required to meet the project objectives was estimated to be *n* = 3949 using a margin of error of 2% and a confidence level of 99%. However, after adjusting the sample size to a 15% response rate, the required sample was *n* = 4195. This sample size gave a greater power. The inclusion criteria included (i) Patients visiting governmental and military hospitals in the north, middle, and south of Jordan, (ii) 18 years or above, (iii) healthy or previously diagnosed with CVD and/or T2D, and (iv) Arabic speaking Jordanians. Exclusion criteria included pregnant females and those who were terminally ill or had dementia, deafness, or mental disorders that hindered the completion of the questionnaire. The diagnosis of T2D among Jordanians is usually based on WHO diagnostic criteria. The diabetes diagnosis is confirmed with (1) fasting plasma glucose ≥ 7.0 mmol/L (126 mg/dL) or (2) 2–h plasma glucose ≥ 11.1 mmol/L (200 mg/dL) after the ingestion of a 75 g oral glucose load. The diagnosis can be confirmed by repeated testing and by using different criteria on different days [28]. CVD was defined as having at least one of the cardiovascular diseases as described by the WHO: (1) Coronary heart disease, (2) Cerebrovascular disease, (3) Peripheral arterial disease, (4) Rheumatic heart disease, (5) Congenital heart disease, (6) Deep vein thrombosis and pulmonary embolism, in addition to heart attacks and strokes as acute events are mainly caused by a blockage that prevents the blood from flowing to the heart or brain [29].

**Figure 1:**
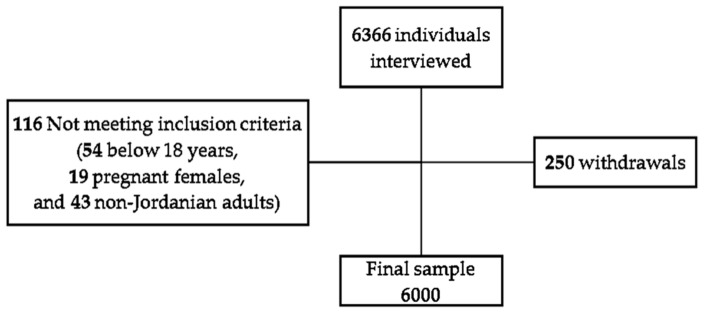
Flow diagram for participant recruitment and procedures for cross-sectional study assessing obesity measures as predictors of type 2 diabetes (T2D) and cardiovascular diseases (CVD) among Jordanian adults.

Out of 6366 individuals interviewed: 116 subjects were excluded (54 young individuals (<18 years), 19 pregnant females, and 43 non-Jordanian adults) and 250 individuals did not complete the questionnaire (response rate of 96.1%) (Figure 1).

The socio-demographic characteristics of the participants, including their age, job nature, education level, sex, and income level, were identified using a questionnaire.

The participants were informed about the purpose and the design of the study and signed a consent form before enrollment. The study protocol was approved by the Institutional Review Board at Jordan University of Science and Technology, the Ministry of Health, and the Royal Medical Services.

### 2.2. Anthropometric Measurements

Weight (kg) was measured to the nearest 0.1 kg using an electronic digital scale (body fat scale GW22029, GoWISE USA, Phoenix, AZ, USA). According to the manual instructions, the scale was calibrated, placed on a hard flat surface, and checked for zero balance before each measurement. The participants were weighed in light clothing and standing-barefoot condition by trained staff using a standard procedure [30]. Height (cm) was measured to the nearest 0.5 cm using a portable Stadiometer (portable mechanical stadiometer HM200P, Charder, Taiwan). For accurate measurement, the participants were barefoot with minimal clothing to facilitate a correct position and having their heels together, arms to the side, legs straight, shoulders relaxed, and the head in the Frankfort horizontal plane [30]. BMI was calculated according to Quetelet’s index: BMI = weight (kg)/height^2^ (m^2^) [31], and categorized according to the WHO criteria: <18.50 kg/m^2^ (underweight), 18.50–24.99 kg/m^2^ (normal), ≥25.00 kg/m^2^ (overweight), and ≥30.00 kg/m^2^ (obese) [10]. WC was measured to the nearest 0.1 cm using a non-elastic measuring tape. The measurement was taken at the narrowest area of the torso between the lower margin of the last palpable rib and the top of the iliac crest [32]. WC > 102 cm in males and WC > 88 cm in females was considered “enlarged WC” according to the WHO cutoff values [10]. These cutoff values were also identified as the cutoffs for the increased relative risk of diseases including T2D and CVD. However, there are ethnic and age-related differences that may affect the predictive validity using these cutoff points [33].

### 2.3. Statistical Analysis

The data were analyzed using a statistical package for social science (SPSS) software (IBM SPSS Statistics for Windows, Version 21.0. Armonk, NY, USA: IBM Corp). Normality was tested, and the results were reported as mean ± SD for normally distributed data sets. The continuous variables were compared using ANOVA. Numbers and frequencies were used to report the categorical variables that were compared using the Chi-square test. Type-I error (false–positive) was avoided by setting the significance level at *p* < 0.05. Type-II error (false negative) was avoided by increasing the statistical power and recruiting a convenient sample size. The risk prediction for the dependent variables (CVD/T2D) by BMI and WC in males, females, and the total population was assessed using the multivariate logistic regression. For regression analysis, the independent variables were dichotomized into two categories: BMI (<25 kg/m^2^ and ≥25 kg/ m^2^) and WC (Normal, Enlarged). The regression analysis included four models: (i) Unadjusted, (ii) Age-adjusted, (iii) Age- and job-nature-adjusted (iv) Age-, job-nature-, education-level-, and income-level-adjusted model. For all variables, the missing values were omitted from the analysis using the stepwise deletion method. The significance for all tests was set at *p* < 0.05.

### 2.4. Institutional Review Board Statement

The study was conducted according to the guidelines laid down in the Declaration of Helsinki, and all procedures involving research study participants were approved by the Institutional Review Board at Jordan University of Science and Technology (61/117/2018) in 22 April 2018.

## 3. Results

### 3.1. BMI Outcomes and Their Relation with Population Demographics

The final sample consisted of 6000 Jordanian adults with a mean age of 41.5 ± 14.7 years and 73.6% of females, of which 48.8% had a medium monthly income, 85.9% had a non-administrative job, and 36.2% had an education level of university degree or higher. In this study, 41.8% of the participants were obese (45.0% in females and 33.0% in males) (Table 1).

The BMI association with the demographic characteristics of the study population was shown in Table 1. BMI was positively associated with the mean age of both sexes (*p* < 0.001). The highest prevalence of obesity was found among the 41–50 age group in males (28.7%) and females (29.7%) compared to other age groups. At the same age period, more participants were obese compared with other BMI categories in both sexes (*p* < 0.001). The income level varied significantly between the four BMI categories in males (*p* = 0.042) and females (*p* < 0.001). However, after applying a Bonferroni correction, the significant differences were only retained between females with obesity and normal-weight females, and between females with obesity and overweight females (*p* < 0.001). There was a higher percentage of obese individuals in the low-income category (33.5% and 46.3%, respectively) compared to other BMI categories. The job nature differed significantly between BMI categories in females (*p* < 0.001) but not in males. More obese females had an education level of high school or below (74.6%), compared to overweight (60.0%), normal-weight (53.3%), and underweight (49.6%) females. However, males’ education level did not vary significantly between different BMI groups.

### 3.2. WC Outcomes and Their Relation with Population Demographics

Among the study population, 44.0% had a large WC size (27.4%% in males and 50.0% in females). Table 2 presents the association between WC and the sample demographics. WC was positively associated with the mean age in male and female participants (*p* < 0.001) and with the highest prevalence of enlarged WC within the age period 41–50 years in males and females (29.4% and 29.9%, respectively) compared to its prevalence in other age categories. More participants had a normal WC between 21 and 30 years in males and females (28.4% and 32.0%, respectively) compared with other age categories (*p* < 0.001). We observed a significant correlation between WC and income level in males (*p* = 0.026) and females (*p* < 0.001). More participants among the low-income category had enlarged WC compared to others with normal WC (males: 35.7% vs. 28.9%; females: 45.9% vs. 37.7%), while other income categories were associated with a higher prevalence of normal WC compared to enlarged WC. The prevalence of enlarged WC varied significantly between different education levels in males (*p* = 0.017) and females (*p* < 0.001).

### 3.3. The Relaltionship of T2D with BMI, WC, and Demographics

The prevalence of T2D was 12.8% (*n* = 765). T2D was positively associated with BMI in both sexes (*p* < 0.001), with its highest prevalence among obese males (45.6%) and females (65.8%) compared with other BMI categories. More participants with obesity had T2D compared to participants with obesity, having normal fasting glucose (NFG) in males (45.6% vs. 31.1%) and females (65.8% vs. 42.0%). The results presented 43.8% of males and 70.4% of females with T2D as having an enlarged WC. In comparison, for NFG participants, we found that 24.9% of males and 47.1% of females had an enlarged WC. As the participants’ age increases, the prevalence of T2D also increased in both sexes (*p* < 0.001), except for females over 60 years as the T2D prevalence decreased. More participants had T2D than others, having NFG in males at 51–60 years (27.2% vs. 13.5%) or older than 60 (44.7% vs. 11.5%) and in females at 51–60 years (46.0% vs. 15.4%) or older than 60 (28.8% vs. 5.7%). The income level varied significantly between T2D and NFG participants in males (*p* = 0.001) and females (*p* < 0.001). The highest prevalence of T2D was found in middle-income and low-income groups in males (40.3% and 39.3%) and females (47.4% and 47.4%). About one-fifth of males with diabetes had a high income level, while only 5.2% of females with diabetes had a high income. Being involved in a non-administrative job was associated with a higher prevalence of T2D in males (86.4% vs. 77.1%, *p* = 0.002) and females (96.1% vs. 87.5%, *p* < 0.001), compared to administrative jobs, which were more associated with having NFG in males (22.9% vs. 13.6%, *p* = 0.002) and females (12.5% vs. 3.9%, *p* < 0.001). The education level varied significantly between T2D and NFG participants in males (*p* = 0.001) and females (*p* < 0.001). A higher proportion of participants with diabetes had an education level of high school or below compared to others with a higher education level in males (72.4% vs. 27.7%) and females (83.7% vs. 16.3%) (Table 3).

### 3.4. The Relationship between BMI, WC, and Demographics and CVD

The prevalence of CVD was 6.3% (*n* = 376). CVD was significantly associated with obesity, as more individuals with obesity had CVD in both males (50.8% vs. 31.5%, *p* < 0.001) and females (67.2% vs. 43.6%, *p* < 0.001) compared to non-CVD individuals. Participants with abdominal obesity, measured by WC, also had higher CVD cases than those with non-CVD in males (52.1% vs. 25.3%, *p* < 0.001) and females (72.7% vs. 48.6%, *p* < 0.001). The prevalence of CVD increased with age, reaching its highest prevalence after 60 years in males (45.8%), and between 51–60 years in females (32.0%), followed by a decreased prevalence after 60 years in females (25.0%). At the age of 51 years and older more males (73.3% vs. 27.6%, *p* < 0.001) and females (57.0% vs. 26.1%, *p* < 0.001) were CVD patients compared to non-CVD cases. In this study, having a low income was associated with a higher presence of CVD in males (39.2% vs. 30.2%, *p* = 0.036) and females (57.4% vs. 40.8%, *p* < 0.001), while other income levels were more associated with CVD absence. Besides, having a low education level (high school and below) was associated with more CVD cases (79.3% vs. 63.8%), while a higher level of education was associated with more non-CVD cases, which was significant only in females (*p* < 0.001) (Table 4).

### 3.5. BMI and WC and Their Association with the Risk of T2D

Table 5 presents the multivariate regression analysis for the association between BMI/WC and T2D among the study population. In the unadjusted model, a BMI of ≥25 kg/m2 was associated with increased odds of having T2D in the total population (OR = 2.9, CI: 2.30–3.80, *p* < 0.001), also when separated by sex, and the risk was higher in females (OR = 3.1, CI: 2.26–4.35, *p* < 0.001) than in males (OR = 2.5, CI: 1.60–3.81, *p* < 0.001). Besides, an enlarged WC was associated with increased odds of having T2D in the total population (OR = 1.7, CI: 1.46–2.06, *p* < 0.001), also in males (OR = 1.8, CI: 1.28–2.44, *p* = 0.001) and females (OR = 1.8, CI: 1.49–2.26, *p* < 0.001), separately. With age-adjustment, the odds of having T2D among the high-BMI and -WC categories decreased, with WC being a significant predictor of T2D among the study population only if not separated by sex. A further adjustment for job did not change the T2D risk among the high-BMI and -WC categories. After adjusting the other demographic variables in the fourth model, a slight reduction was seen in the risk of T2D among the high BMI group in the total population and in the risk of T2D among high BMI and WC groups in females. In the final model, the BMI (OR = 1.7, CI: 1.30–2.30, *p* < 0.001) predicted a higher risk of T2D compared to WC (OR = 1.3, CI: 1.04–1.52, *p* = 0.016) in the total population. Moreover, BMI was the only predictor of T2D in both males (OR = 1.8, CI: 1.15–2.94, *p* = 0.011) and females (OR = 1.6, CI: 1.13–2.33, *p* = 0.008).

### 3.6. BMI and WC and Their Association with the Risk of CVD

The multivariate regression analysis for the association between BMI/WC and CVD among the study population was shown in Table 6. In the unadjusted model, the enlarged WC was associated with increased odds of CVD in the total population (OR = 2.5, CI: 1.93–3.20, *p* < 0.001), also when separated by sex, and the risk was higher in males (OR = 3.0, CI: 1.95–4.53, *p* < 0.001) than in females (OR = 2.8, CI: 1.98–3.84, *p* < 0.001). With age-adjustment, the odds ratio of having CVD among the high WC category decreased in the total population, and in males and females separately. A further adjustment for job did not change the CVD risk in the high-WC group. After adjusting the other demographic variables in the fourth model, a slight reduction was seen in the risk of CVD among the high-WC group in the total population (OR = 1.9, CI: 1.47–2.47, *p* < 0.001) and when separated by sex (Males: OR = 2.2, CI: 1.45–1.48, *p* < 0.001) and (Females: OR = 2.1, CI: 1.48–2.92, *p* < 0.001)). In all regression models, the BMI was not a predictor for CVD risk in the total population and when separated by sex.

## 4. Discussion

Our results presented BMI as a better predictor for T2D than WC, while WC was a better predictor for CVD than BMI among Jordanian adults. The suitability of different anthropometric measurements and prediction models for predicting chronic diseases among Jordanian and Arab adults might differ from that of other populations due to ethnic differences [10,34].

In this nationwide study, the obesity prevalence was 41.8%, with 45.0% in females and 33.0% in males. Our results were consistent with a previous study in Jordan reporting a high obesity prevalence wherein 36.1% of males and 48.2% of females had obesity [35], but it is higher than the prevalence reported by WHO in 2016, which was 21.0% in males and 35.6% in females [6].

In this study, the prevalence of obesity was positively associated with age in males and females, with its highest prevalence among participants with 41–50 years. By comparison, in Iran, another country in the Middle East, the differences in the prevalence of obesity between age groups were not significant. Yet, the highest prevalence was associated with being 55–59 years old [36]. In Norway, the highest prevalence of obesity according to age group differed between sexes, as it was between 70 and 74 years in women and between 45 and 49 years in men. The prevalence of abdominal obesity was also higher in the elderly compared to young adults in Norway and Portugal [37,38], which was consistent with our results. In China, older individuals were more likely to have a higher WC than younger adults [39]. In this study, we found that low income and low education levels might be associated with increased obesity and abdominal obesity among the study population. Similarly, in Iran, obesity prevalence was associated with low education levels, wherein the illiterate were more obese than individuals with college education [36]. A lower educational level was also associated with a higher prevalence of obesity and abdominal obesity in Portugal [38]. In China, individuals with moderate, moderate-high, or high WC had a low education but a high-income level than others with low WC within a long time interval [39]. Another study across low- and middle-income countries showed that obesity prevalence might increase with increasing education, and it was positively associated with the country’s income level. Further, the study reported a stronger association between increased BMI and diabetes when the country’s income level increased [40].

The rising prevalence of overnutrition is alarming and needs a serious intervention due to the strong association between adiposity, obesity, and central obesity with CVD and T2D [10]. As shown in this study, the prevalence of CVD and T2D was higher among participants who had obesity or central obesity, which is consistent with the literature [39,41,42]. Studies from the U.S. indicated that adults having a BMI above the normal (over 24.9 kg/m^2^) were at increased risk of developing CVD [43] and T2D [44]. A Korean study reported an increased risk of CVD among male and female adults with a BMI of ≥26.2 and ≥28.7 kg/m^2^, respectively. For T2D, the increased risk of disease was associated with a BMI of ≥24.2 in men and ≥23.6 in women [45]. The increase in central obesity, measured by WC, was associated with a 2% increase in the relative risk of CVD and an 8% increase in the relative risk of T2D with each unit increase in WC [46,47].

CVD are the major contributors to deaths from non-communicable diseases worldwide, followed by cancer, respiratory diseases, and DM [48]. An estimated 17.9 million people died from CVD in 2019, representing 32% of all global deaths. Of these deaths, 85% were due to heart attack and stroke [29]. In 2019, DM was the ninth leading cause of death, with an estimated 1.5 million deaths directly caused by DM. The prevalence has been rising more rapidly in low- and middle-income countries than in high-income countries [49]. CVD is also the main contributor to premature deaths, and it accounts for 22% of all deaths in the European Union [50]. In 2016, the WHO reported that CVD and T2D accounted for 37% and 6% of all deaths among Jordanians [27]. A systematic review for the prevalence of CVD and T2D, as one of the CVD’s risk factors, in some countries of the Middle East showed that CVD prevalence was 10.1% and the prevalence of DM was 16% [51]. However, no representative study showed the prevalence of at-risk or existing CVD among Jordanians [27], while the WHO estimated the prevalence of DM in 2014 to be 13% [48].

Our study showed that the prevalence of CVD and T2D was 6.3% and 12.8%, respectively. A recent review showed that the prevalence of some types of CVD among Lebanese men was 10.1% for myocardial infarction and 8.2% for angina, while the prevalence of total coronary artery disease was 13.4% for adults above 40 years [52]. CVD prevalence among Jordanians was comparable to its prevalence in Lebanon (6.5%) in 2014. However, the prevalence was lower than the prevalence in other Middle Eastern countries, including Oman (9.4%) and Iran (11.2%) in 2018, and higher than the prevalence in Turkey in 2015 [51]. The prevalence of DM was consistent with previous data provided by the international DM federation (11.8%) in 2017. The DM prevalence in Jordan was comparable to its prevalence in other nearby countries such as Lebanon (12.7%), lower than its prevalence in Egypt (17.3%) and Kuwait (15.8%), and higher than its prevalence in Iraq (8.8%) and Iran (9.6%) [53]. Diabetes in Jordan might be compromised by the lack of national diabetic-specific dietary guidelines, poor self-management, and poor blood sugar monitoring among Jordanians with diabetes [54].

We also found that the increase in age was associated with the increased prevalence of T2D, which is consistent with previous studies that emphasized the role of age as a significant risk for T2D among the study population [55,56]. Our results also presented an association between the presence of T2D and low income or low education level. The results were consistent with the previous study in Sweden, as low education and low income levels were linked to elevated glycosylated hemoglobin (>70 mmol/mol or 8.6%) [57]. In Korea, low education and low income contributed to a higher T2D prevalence, although the income level was not an important predictor for T2D among Korean males [58]. Another study in South Africa showed that T2D risk was higher in less educated individuals, while there were no differences based on the income level [56]. We indicated an association between non-administrative jobs and the increased prevalence of T2D. In contrast, holding an administrative position among Japanese males was associated with 12.7 increased odds of developing T2D in males with impaired fasting glucose or impaired glucose tolerance [59].

The results presented older adults with a higher prevalence of CVD, which decreased after reaching 60 years only in females. Our results agreed with a study among low- and middle-income countries showing that increasing age was associated with an increased incidence of some CVD, namely angina and stroke, which was followed by a decreased risk in the elderly in some countries [60]. At the age of 51 years and older, more males (73.3% vs. 27.6%, *p* < 0.001) and females (57.0% vs. 26.1%, *p* < 0.001) were CVD patients compared to non-CVD cases. The low education in females and the low income among the study population were also associated with an increased prevalence of CVD. Likewise, in Sweden, the prevalence of ischemic heart disease was higher in low-income and low-education-level groups [57]. A previous study across 20 countries with different income levels revealed that individuals with an education level of primary school or below had a higher chance of developing CVD than others with a university degree or above [61]. However, a study from India showed that rich households and secondary school levels were more associated with a higher CVD risk than other household wealth and education categories [62]. Our analysis also represented a higher presence of CVD among participants enrolled in non-administrative jobs, which agreed with a previous study in Turkey [63]. By comparison, in Japan, the managers and professors (high occupational levels) were more likely to have an increased risk of coronary heart disease but a lower risk of stroke [64].

In this study, BMI was a better predictor for T2D compared to WC in males and females. In comparison, in China, BMI was defined as the best indicator for the association between obesity and T2D in women but not in men [65]. Another study in China suggested that WC was a better predictor for T2D compared to BMI, and WC predicted a higher risk of metabolic syndrome and dyslipidemia [66,67]. Compared to BMI, WC was associated with T2D and several markers of insulin resistance in French women having severe obesity [68]. Another study found that BMI was not associated with FBG, glycosylated hemoglobin, insulin resistance, and hyperinsulinemia in South Africa [56]. A previous study in two European countries showed that BMI and WC were associated with T2D, with a higher OR of having T2D within the highest WC group than the highest BMI group [69]. In a Europe-wide study, Feller and colleagues reported that the association of WC with T2D might decrease at a BMI level of >30 kg/m^2^; however, both BMI and WC were associated with T2D risk [47]. An analysis of 16 cohort studies from seven countries in Asia showed that the association between obesity indicators and T2D varied with age, and that T2D had a stronger association with waist-stature-ratio than BMI in individuals under 50 years, while the association between T2D and central obesity indicators, including WC, did not differ from BMI in individuals ≥50 years. Nevertheless, in the age- and study-cohort-adjusted model, the association of T2D with WC was more robust than its association with BMI only in women [70].

Our results proposed WC as a better predictor for CVD than BMI, underlining the fact that central obesity might be associated with increased risk of CVD even in normal-weight individuals [71]. A cohort study among the U.S. population showed that BMI and WC were strong predictors for disease comorbidities when used separately; nonetheless, only WC remained a predictor for cardiometabolic risk when both indicators were incorporated in the prediction model [72]. Another study from India showed that CVD was significantly correlated with BMI, WC, and waist-to-height ratio. However, the negative correlation between high-density lipoprotein, a CVD risk indicator, was strongest with WC compared to other anthropometric measurements [73]. The BMI predicted a higher risk of hypertension, a CVD risk factor, compared to WC among the Chinese population [66]. In contrast, a study showed that in women with severe obesity, WC had a stronger association with hypertension and several markers of cardiometabolic risk factors in France [68]. Likewise, in Spain, WC was considered the best predictor of cardiometabolic risk in individuals with morbid obesity [74].

Our results showed a trend of decreased CVD risk with a BMI ≥ 25 after adjusting the demographic variables. A Previous study showed that underweight is an independent risk factor for CVD, particularly in adults below 60 years [75]. Another study among elderly showed that both underweight and morbidly obesity (BMI ≥ 35) are associated with CVD, and expanded CVD mortality [76]. Malnutrition through early life-stages might have an effect on the risk of CVD among adults. For example, a study showed that adults who survived after a severe acute malnutrition during childhood are at higher risk of impaired cardiovascular and metabolic function that might be related to impaired cardiovascular development, elevated peripheral resistance, and diastolic blood pressure [77]. A review of 57 studies, showed that the exposure to famine or severe malnutrition in childhood is associated with increased risk of CVD and other metabolic abnormalities [78].

While the results from this study can be applied to other nearby Arabic countries sharing similar backgrounds with Jordan, our study is limited by the lack of other anthropometric indices that can be used in risk prediction. The WC cutoff points in this study are based on the WHO recommendations, which are undefined for the Jordanian population. Furthermore, our results are based on a cross-sectional design that can provide a possible association or evidence, but they are not conclusive. Hence, the results may not be generalized and should be treated cautiously. The ages of the study population ranged from 18 years to 92 years, and this wide range is the main reason for the large SD. This might be one of the limitations of our study.

## 5. Conclusions

In conclusion, the prevalence of CVD and T2D was 6.3% and 12.8%, respectively. Our study helps identify the risk of T2D and CVD among the Jordanian population. We emphasized that disease prediction needs a careful selection of the anthropometric indices that might affect the prediction models outcomes in a selected population. In this study, we find that BMI predicts a higher risk of T2D, and it is more suitable for risk prediction in males and females, separately. On the other hand, WC is more efficient in predicting CVD risk among Jordanians. As our results differ from studies from other non-Arabic countries, we emphasize that future research on disease prediction should consider the population-based differences. More research is needed to identify the ability of other anthropometric indices to assess the risk of T2D and CVD and to determine the suitability of the currently used cut-off point for the Jordanian population.

## Figures and Tables

**Table 1:** BMI relation with population characteristics stratified by sex ^1^.

Variable	Total	Male				*p*-Value	Female				*p*-Value
		Under wt	Normal wt	Over wt	Obese		Under wt	Normal wt	Over wt	Obese	
Age (mean ± SD)	41.5 ± 14.7	30.0 ± 13.6	35.8 ± 16.2	43.0 ± 16.0	47.9 ± 14.6	<0.001	28.0 ± 9.8	33.0 ± 12.6	40.6 ± 13.6	47.0 ± 12.4	<0.001
Age											
≤20	389 (6.5)	9 (25.7)	71 (15.3)	25 (4.5)	14 (2.7)	<0.001	25 (21.4)	141 (13.0)	70 (5.7)	34 (1.7)	<0.001
21–30	1309 (21.8)	14 (40.0)	171 (36.8)	121 (21.6)	63 (12.1)		62 (53.0)	435 (40.0)	256 (20.9)	187 (9.4)	
31–40	1183 (19.7)	4 (11.4)	71 (15.3)	119 (21.3)	77 (14.8)		17 (14.5)	237 (21.8)	304 (24.8)	354 (17.8)	
41–50	1394 (23.2)	6 (17.1)	55 (11.8)	120 (21.4)	150 (28.7)		9 (7.7)	168 (15.4)	296 (24.2)	590 (29.7)	
51–60	1094 (18.2)	0 (0.0)	47 (10.1)	85 (15.2)	110 (21.1)		2 (1.7)	69 (6.3)	201 (16.4)	580 (29.2)	
>60	631 (10.5)	2 (5.7)	50 (10.8)	90 (16.1)	108 (20.7)		2 (1.7)	38 (3.5)	98 (8.0)	243 (12.2)	
Income Level (JOD) ^2^											
<350	2332 (38.9)	8 (22.9)	134 (28.8)	171 (30.5)	175 (33.5)	0.042	47 (40.2)	401 (36.9)	475 (38.8)	921 (46.3)	<0.001
350–799	2926 (48.8)	16 (45.7)	247 (53.1)	285 (50.9)	278 (53.3)		58 (49.6)	527 (48.4)	611 (49.9)	904 (45.5)	
≥800	742 (12.4)	11 (31.4)	84 (18.1)	104 (18.6)	69 (13.2)		12 (10.3)	160 (14.7)	139 (11.3)	163 (8.2)	
Job Nature											
Administrative	846 (14.1)	6 (17.1)	86 (18.5)	122 (21.8)	129 (24.7)	0.11	9 (7.7)	139 (12.8)	171 (14.0)	184 (9.3)	<0.001
Non-administrative	5154 (85.9)	29 (82.9)	379 (81.5)	438 (78.2)	393 (75.3)		108 (92.3)	949 (87.2)	1054 (86.0)	1804 (90.7)	
Educational Level											
<High School	437 (7.3)	1 (2.9)	27 (5.8)	31 (5.5)	40 (7.7)	0.082	5 (4.3)	60 (5.5)	96 (7.8)	177 (8.9)	<0.001
High School	3396 (56.6)	20 (57.1)	242 (52.0)	308 (55.0)	307 (58.8)		53 (45.3)	520 (47.8)	639 (52.2)	1307 (65.7)	
University	2019 (33.7)	14 (40.0)	184 (39.6)	196 (35.0)	158 (30.3)		56 (47.9)	481 (44.2)	459 (37.5)	471 (23.7)	
Postgraduate	148 (2.5)	0 (0.0)	12 (2.6)	25 (4.5)	17 (3.3)		3 (2.6)	27 (2.5)	31 (2.5)	33 (1.7)	

^1^ Data are presented as number of observations and frequencies (*n* (%)) unless otherwise indicated. The differences in the sociodemographic characteristics between weight categories were assessed by ANOVA for continuous variables and by χ2 test for categorical variables. *p*-values < 0.05 was considered significant for all statistical analyses. ^2^ JOD: Jordanian Dinar.

**Table 2:** Waist Circumference (WC) relation with population characteristics stratified by sex ^1^.

Variable	Total ^2^	Male		*p*-Value	Female		*p*-Value
		Enlarged WC ^2^	Normal WC		Enlarged WC	Normal WC	
Age (mean ± SD)		49.3 ± 14.3	39.5 ± 16.3	<0.001	46.4 ± 12.5	36.2 ± 13.7	<0.001
Age							
≤20	384 (6.5)	6 (1.4)	111 (9.8)	<0.001	38 (1.8)	229 (10.6)	<0.001
21–30	1288 (21.8)	44 (10.3)	323 (28.4)		227 (10.5)	694 (32.0)	
31–40	1161 (19.7)	61 (14.3)	206 (18.1)		407 (18.8)	487 (22.4)	
41–50	1375 (23.3)	126 (29.4)	200 (17.6)		648 (29.9)	401 (18.5)	
51–60	1075 (18.2)	92 (21.5)	149 (13.1)		596 (27.5)	238 (11.0)	
>60	621 (10.5)	99 (23.1)	147 (12.9)		254 (11.7)	121 (5.6)	
Income Level (JOD) ^3^							
<350	2296 (38.9)	153 (35.7)	328 (28.9)	0.026	997 (45.9)	818 (37.7)	<0.001
350–799	2878 (48.7)	204 (47.7)	615 (54.1)		990 (45.6)	1069 (49.3)	
≥800	730 (12.4)	71 (16.6)	193 (17.0)		183 (8.4)	283 (13.0)	
Job Nature							
Administrative	837 (14.2)	105 (24.5)	236 (20.8)	0.114	233 (10.7)	263 (12.1)	0.166
Non-administrative	5067 (85.8)	323 (75.5)	900 (79.2)		1937 (89.3)	1907 (87.9)	
Educational Level							
<High School	433 (7.3)	23 (5.4)	76 (6.7)	0.017	195 (9.0)	139 (6.4)	<0.001
High School	3338 (56.5)	255 (59.6)	610 (53.7)		1392 (64.1)	1081 (49.8)	
University	1985 (33.6)	129 (30.1)	417 (36.7)		548 (25.3)	891 (41.1)	
Postgraduate	148 (2.5)	21 (4.9)	33 (2.9)		35 (1.6)	59 (2.7)	

^1^ Data are presented as number of observations and frequencies (*n* (%)) unless otherwise indicated. The differences in the sociodemographic characteristics between WC categories were assessed by ANOVA for continuous variables and by χ2 test for categorical variables. *p*-values < 0.05 was considered significant for all statistical analyses. ^2^ The total number of observations is *n* = 5904, the WC was missing for 96 participants. ^3^ JOD: Jordanian Dinar, WC: Waist Circumference.

**Table 3:** Type 2 Diabetes (T2D) relation with BMI, WC, and population characteristics stratified by sex ^1^.

Variable	Total	Male		*p*-Value	Female		*p*-Value
		NFG ^2^	T2D ^2^		NFG	T2D	
BMI Classification							
Underweight	152 (2.53)	35 (2.5)	0 (0.0)	<0.001	116 (3.0)	1 (0.2)	<0.001
Normal weight	1553 (25.9)	436 (31.7)	29 (14.1)		1037 (26.9)	51 (9.1)	
Overweight	1785 (29.8)	477 (34.7)	83 (40.3)		1086 (28.1)	139 (24.9)	
Obese	2510 (41.8)	428 (31.1)	94 (45.6)		1620 (42.0)	368 (65.8)	
WC ^3^							
Normal	3306 (56.0)	1022 (75.1)	114 (56.2)	<0.001	2008 (52.9)	162 (29.6)	<0.001
Enlarged	2598 (44.0)	339 (24.9)	89 (43.8)		1785 (47.1)	385 (70.4)	
Age							
≤20	389 (6.5)	119 (8.6)	0 (0.0)	<0.001	270 (7.0)	0 (0.0)	<0.001
21–30	1309 (21.8)	364 (26.5)	5 (2.4)		927 (24.0)	13 (2.3)	
31–40	1183 (19.7)	261 (19.0)	10 (4.9)		875 (22.7)	37 (6.6)	
41–50	1394 (23.2)	288 (20.9)	43 (20.9)		972 (25.2)	91 (16.3)	
51–60	1094 (18.2)	186 (13.5)	56 (27.2)		595 (15.4)	257 (46.0)	
>60	631 (10.5)	158 (11.5)	92 (44.7)		220 (5.7)	161 (28.8)	
Income Level (JOD) ^4^							
<350	2332 (38.9)	407 (29.6)	81 (39.3)	0.001	1579 (40.9)	265 (47.4)	<0.001
350–799	2926 (48.8)	743 (54.0)	83 (40.3)		1835 (47.6)	265 (47.4)	
≥800	742 (12.4)	226 (16.4)	42 (20.4)		445 (11.5)	29 (5.2)	
Job Nature							
Administrative	846 (14.1)	315 (22.9)	28 (13.6)	0.002	481 (12.5)	22 (3.9)	<0.001
Non-administrative	5154 (85.9)	1061 (77.1)	178 (86.4)		3378 (87.5)	537 (96.1)	
Educational Level							
<High School	437 (7.3)	76 (5.5)	23 (11.2)	0.001	259 (6.7)	79 (14.1)	<0.001
High School	3396 (56.6)	751 (54.6)	126 (61.2)		2130 (55.2)	389 (69.6)	
University	2019 (33.7)	501 (36.4)	51 (24.8)		1378 (35.7)	89 (15.9)	
Postgraduate	148 (2.5)	48 (3.5)	6 (2.9)		92 (2.4)	2 (0.4)	

^1^ Data are presented as number of observations and frequencies (*n* (%)). The differences between the individuals having normal fasting glucose (NFG) and individuals having T2D were assessed by a χ2 test for categorical variables. *p*-values < 0.05 was considered significant for all statistical analyses. *p*-values < 0.05 was considered significant for all statistical analyses. ^2^ NFG: Normal Fasting Glucose, T2D: Type 2 Diabetes. ^3^ The total number of observations is *n* = 5904, the WC was missing for 96 participants. ^4^ JOD: Jordanian Dinar.

**Table 4:** CVD relation with BMI, WC, and population characteristics stratified by sex ^1^.

Variable	Total	Male		*p*-Value	Female		*p*-Value
		Presence ^2^	Absence ^2^		Presence	Absence	
BMI Classification							
Underweight	152 (2.53)	0 (0.0)	35 (2.4)	<0.001	1 (0.4)	116 (2.8)	<0.001
Normal weight	1553 (25.9)	24 (20.0)	441 (30.2)		43 (16.8)	1045 (25.1)	
Overweight	1785 (29.8)	35 (29.2)	525 (35.9)		40 (15.6)	1185 (28.5)	
Obese	2510 (41.8)	61 (50.8)	461 (31.5)		172 (67.2)	1816 (43.6)	
WC ^3^							
Normal	3306 (56.0)	57 (47.9)	1079 (74.7)	<0.001	68 (27.3)	2102 (51.4)	<0.001
Enlarged	2598 (44.0)	62 (52.1)	366 (25.3)		181 (72.7)	1989 (48.6)	
Age							
≤20	389 (6.5)	4 (3.3)	115 (7.9)	<0.001	3 (1.20)	267 (6.40)	<0.001
21–30	1309 (21.8)	5 (4.2)	364 (24.9)		21 (8.20)	919 (22.1)	
31–40	1183 (19.7)	8 (6.7)	263 (18.0)		34 (13.3)	878 (21.1)	
41–50	1394 (23.2)	15 (12.5)	316 (21.6)		52 (20.3)	1011 (24.3)	
51–60	1094 (18.2)	33 (27.5)	209 (14.3)		82 (32.0)	770 (18.5)	
>60	631 (10.5)	55 (45.8)	195 (13.3)		64 (25.0)	317 (7.6)	
Income Level (JOD) ^4^							
<350	2332 (38.9)	47 (39.2)	441 (30.2)	0.036	147 (57.4)	1697 (40.8)	<0.001
350–799	2926 (48.8)	61 (50.8)	765 (52.3)		96 (37.5)	2004 (48.1)	
≥800	742 (12.4)	12 (10.0)	256 (17.5)		13 (5.10)	461 (11.1)	
Job Nature							
Administrative	846 (14.1)	15 (12.5)	328 (22.4)	0.011	12 (4.70)	491 (11.8)	<0.001
Non-administrative	5154 (85.9)	105 (87.5)	1134 (77.6)		244 (95.3)	3671 (88.2)	
Educational Level							
<High School	437 (7.3)	6 (5.0)	93 (6.4)	0.116	26 (10.2)	312 (7.5)	<0.001
High School	3396 (56.6)	79 (65.8)	798 (54.6)		177 (69.1)	2342 (56.3)	
University	2019 (33.7)	31 (25.8)	521 (35.6)		53 (20.7)	1414 (34.0)	
Postgraduate	148 (2.5)	4 (3.3)	50 (3.4)		0 (0.0)	94 (2.3)	

^1^ Data are presented as number of observations and frequencies (*n* (%)). The differences between the individuals having CVD and the individuals without CVD were assessed by a χ2 test for categorical variables. *p*-values < 0.05 was considered significant for all statistical analyses. *p*-values < 0.05 was considered significant for all statistical analyses. ^2^ Presence: Diagnosed with CVD, Absence: Undiagnosed with CVD. ^3^ The total number of observations is *n* = 5904, the WC was missing for 96 participants. ^4^ JOD: Jordanian Dinar.

**Table 5:** Associations of body mass index (BMI) and WC with T2D among the study population stratified by sex ^1^.

Model	Variable	All Population	*p*-Value	Male	*p*-Value	Female	*p*-Value
		OR (95% CI)		OR (95% CI)		OR (95% CI)	
Model # 1							
	BMI classification						
	<25 kg/m^2^	Reference	<0.001	Reference	<0.001	Reference	<0.001
	≥25 kg/m^2^	2.9 (2.30–3.80)		2.5 (1.60–3.81)		3.1 (2.26–4.35)	
	WC ^2^						
	Normal	Reference	<0.001	Reference	0.001	Reference	<0.001
	Enlarged	1.7 (1.46–2.06)		1.8 (1.28–2.44)		1.8 (1.49–2.26)	
Model # 2							
	BMI classification						
	<25 kg/m^2^	Reference	<0.001	Reference	0.014	Reference	0.005
	≥25 kg/m^2^	1.8 (1.32–2.32)		1.8 (1.12–2.85)		1.7 (1.17–2.39)	
	WC						
	Normal	Reference	0.007	Reference	0.117	Reference	0.059
	Enlarged	1.3 (1.07–1.56)		1.3 (0.90–1.81)		1.3 (0.99–1.58)	
Model # 3							
	BMI classification						
	<25 kg/m^2^	Reference	<0.001	Reference	0.013	Reference	0.005
	≥25 kg/m^2^	1.8 (1.32–2.34)		1.8 (1.14–2.88)		1.7 (1.17–2.38)	
	WC						
	Normal	Reference	0.010	Reference	0.167	Reference	0.061
	Enlarged	1.3 (1.06–1.55)		1.3 (0.90–1.82)		1.3 (0.99–1.57)	
Model # 4							
	BMI classification						
	<25 kg/m^2^	Reference	<0.001	Reference	0.011	Reference	0.008
	≥25 kg/m^2^	1.7 (1.30–2.30)		1.8 (1.15–2.94)		1.6 (1.13–2.33)	
	WC						
	Normal	Reference	0.016	Reference	0.18	Reference	0.109
	Enlarged	1.3 (1.04–1.52)		1.3 (0.89–1.82)		1.2 (0.96–1.53)	

^1^ The risk prediction for T2D (dependent variable) by BMI and WC in males, females, and the total population was assessed using the multivariate logistic regression. For regression analysis, the independent variables were dichotomized into 2 categories as follows: BMI (<25 kg/m^2^ and ≥25 kg/m^2^) and WC (Normal, Enlarged). The regression analysis included four models: Model 1: Unadjusted, Model 2: Adjusted for age, Model 3: Model 2 + job nature, Model 4: Model 3 + educational level + income level. For all variables, the missing values were omitted from the analysis using the stepwise deletion method. *p*-values < 0.05 was considered significant for all statistical analyses. ^2^ WC: Waist Circumference.

**Table 6:** Associations of BMI and WC with cardiovascular diseases (CVD) among the study population stratified by sex ^1^.

Model	Variable	All Population	*p*-Value	Male	*p*-Value	Female	*p*-Value
		OR (95% CI)		OR (95% CI)		OR (95% CI)	
Model # 1							
	BMI classification						
	<25 kg/m^2^	Reference	0.383	Reference	0.44	Reference	0.818
	≥25 kg/m^2^	1.2 (0.84–1.57)		1.2 (0.73–2.07)		1.1 (0.71–1.55)	
	WC ^2^						
	Normal	Reference	<0.001	Reference	<0.001	Reference	<0.001
	Enlarged	2.5 (1.93–3.20)		3.0 (1.95–4.53)		2.8 (1.98–3.84)	
Model # 2							
	BMI classification						
	<25 kg/m^2^	Reference	0.078	Reference	0.595	Reference	0.079
	≥25 kg/m^2^	0.8 (0.54–1.03)		0.9 (0.50–1.48)		0.7 (0.46–1.04)	
	WC						
	Normal	Reference	<0.001	Reference	<0.001	Reference	<0.001
	Enlarged	2.0 (1.55–2.59)		2.3 (1.50–3.56)		2.2 (1.54–3.01)	
Model # 3							
	BMI classification						
	<25 kg/m^2^	Reference	0.087	Reference	0.642	Reference	0.079
	≥25 kg/m^2^	0.8 (0.54–1.04)		0.9 (0.51–1.51)		0.7 (0.46–1.04)	
	WC						
	Normal	Reference	<0.001	Reference	<0.001	Reference	<0.001
	Enlarged	2.0 (1.53–2.57)		2.3 (1.51–3.61)		2.2 (1.54–3.02)	
Model # 4							
	BMI classification						
	<25 kg/m^2^	Reference	0.75	Reference	0.631	Reference	0.066
	≥25 kg/m^2^	0.7 (0.54–1.03)		0.9 (0.51–1.51)		0.7 (0.45–1.03)	
	WC						
	Normal	Reference	<0.001	Reference	<0.001	Reference	<0.001
	Enlarged	1.9 (1.47–2.47)		2.2 (1.45–1.48)		2.1 (1.48–2.92)	

^1^ The risk prediction for CVD (dependent variable) by BMI and WC in males, females, and the total population was assessed using the multivariate logistic regression. For regression analysis, the independent variables were dichotomized into 2 categories as follows: BMI (<25 kg/m^2^ and ≥25 kg/m^2^) and WC (Normal, Enlarged). The regression analysis included four models: Model 1: Unadjusted, Model 2: Adjusted for age, Model 3: Model 2 + job nature, Model 4: Model 3 + educational level + income level. For all variables, the missing values were omitted from the analysis using the stepwise deletion method. *p*-values < 0.05 was considered significant for all statistical analyses. ^2^ WC: Waist Circumference.

## Data Availability

The data described in the manuscript, code book, and analytic code will be made available upon request pending approval by the authors.

## References

[1] World Health Organization Obesity and Overweight. https://www.who.int/news-room/fact-sheets/detail/obesity-and-overweight.

[2] Hales C., Carroll M., Fryar C., Ogden C. (2020). Prevalence of Obesity and Severe Obesity among Adults: United States, 2017–2018.

[3] World Health Organization European Health Information Gateway, Data, Indicators, Obesity. https://gateway.euro.who.int/en/indicators/h2020_9-obesity/.

[4] Hoffman D.J. (2001). Obesity in developing countries: Causes and implications. Food Nutr. Agric..

[5] Nikoloski Z., Williams G. (2016). Obesity in the Middle East.

[6] World Health Organization Jordan-WHO, Country Profiles. https://www.who.int/diabetes/country-profiles/jor_en.pdf.

[7] Centers for Disease Control and Prevention Adult Obesity Causes and Cosequences. https://www.cdc.gov/obesity/adult/causes.html.

[8] Al-Goblan A.S., Al-Alfi M.A., Khan M.Z. (2014). Mechanism linking diabetes mellitus and obesity. Diabetes Metab. Syndr. Obes. Targets Ther..

[9] Piché M.-E., Tchernof A., Després J.-P. (2020). Obesity phenotypes, diabetes, and cardiovascular diseases. Circ. Res..

[10] World Health Organization (2011). Waist Circumference and Waist-Hip Ratio: Report of a WHO Expert Consultation, Geneva, 8–11 December 2008. https://www.who.int/publications/i/item/9789241501491.

[11] Collins G.S., Mallett S., Omar O., Yu L.-M. (2011). Developing risk prediction models for type 2 diabetes: A systematic review of methodology and reporting. BMC Med..

[12] Qiao Q., Nyamdorj R. (2010). Is the association of type II diabetes with waist circumference or waist-to-hip ratio stronger than that with body mass index?. Eur. J. Clin. Nutr..

[13] Hippisley-Cox J., Coupland C., Vinogradova Y., Robson J., May M., Brindle P. (2007). Derivation and validation of QRISK, a new cardiovascular disease risk score for the United Kingdom: Prospective open cohort study. BMJ.

[14] Cooney M.T., Dudina A.L., Graham I.M. (2009). Value and limitations of existing scores for the assessment of cardiovascular risk: A review for clinicians. J. Am. Coll. Cardiol..

[15] Emerging Risk Factors Collaboration (2011). Separate and combined associations of body-mass index and abdominal adiposity with cardiovascular disease: Collaborative analysis of 58 prospective studies. Lancet.

[16] World Health Organization World Health Organization/International Society of Hypertension (WH0/ISH) Risk Prediction Charts for 14 WHO Epidemiological Sub-Regions (Charts in Colour). https://www.who.int/ncds/management/WHO_ISH_Risk_Prediction_Charts.pdf?ua=1.

[17] Carbone S., Canada J.M., Billingsley H.E., Siddiqui M.S., Elagizi A., Lavie C.J. (2019). Obesity paradox in cardiovascular disease: Where do we stand?. Vasc. Health Risk Manag..

[18] National Heart Lung and Blood Institute Assessing Your Weight and Health Risk. https://www.nhlbi.nih.gov/health/educational/lose_wt/risk.htm.

[19] Bhupathiraju S.N., Hu F.B. (2016). Epidemiology of obesity and diabetes and their cardiovascular complications. Circ. Res..

[20] Darsini D., Hamidah H., Notobroto H.B., Cahyono E.A. (2020). Health risks associated with high waist circumference: A systematic review. J. Public Health Res..

[21] World Health Organization Global Health Risks: Mortality and Burden of Disease Attributable to Selected Major Risks. https://www.who.int/healthinfo/global_burden_disease/GlobalHealthRisks_report_full.pdf.

[22] Kashiwagi R., Iwahashi H., Yamada Y., Sakaue T., Okita T., Kawachi Y., Iwamoto R., Saisho K., Tamba S., Yamamoto K. (2017). Effective waist circumference reduction rate necessary to avoid the development of type 2 diabetes in Japanese men with abdominal obesity. Endocr. J..

[23] American Diabetes Association (2016). 6. Obesity management for the treatment of type 2 diabetes. Diabetes Care.

[24] Haase C.L., Lopes S., Olsen A.H., Satylganova A., Schnecke V., McEwan P. (2021). Weight loss and risk reduction of obesity-related outcomes in 0.5 million people: Evidence from a UK primary care database. Int. J. Obes..

[25] Fanghänel G., Sánchez-Reyes L., Félix-García L., Violante-Ortiz R., Campos-Franco E., Alcocer L.A. (2011). Impact of waist circumference reduction on cardiovascular risk in treated obese subjects. Cir. Cir..

[26] Department of Statistics Population. http://dosweb.dos.gov.jo/ar/population/population-2/.

[27] World Health Organization Risk of Premature Death Due to Ncds, Jordan. https://www.who.int/nmh/countries/jor_en.pdf.

[28] World Health Organization (2006). Definition and Diagnosis of Diabetes Mellitus and Intermediate Hyperglycaemia: Report of a WHO/IDF Consultation.

[29] World Health Organization Cardiovascular Diseases (CVDs). https://www.who.int/news-room/fact-sheets/detail/cardiovascular-diseases-(cvds).

[30] Lee R.D., Nieman D.C. (2010). Nutritional Assessment.

[31] Deurenberg P., Weststrate J.A., Seidell J.C. (1991). Body mass index as a measure of body fatness: Age-and sex-specific prediction formulas. Br. J. Nutr..

[32] Lee R., Lee R.D., Nieman D.C. (2013). Biochemical assessment of nutritional status. Nutritional Assessment: Ney York.

[33] North American Association for the Study of Obesity, National Heart, Lung, Blood Institute, NHLBI Obesity Education Initiative (2000). The Practical Guide: Identification, Evaluation, and Treatment of Overweight and Obesity in Adults.

[34] Lacy M.E., Wellenius G.A., Carnethon M.R., Loucks E.B., Carson A.P., Luo X., Kiefe C.I., Gjelsvik A., Gunderson E.P., Eaton C.B. (2016). Racial differences in the performance of existing risk prediction models for incident type 2 diabetes: The CARDIA study. Diabetes Care.

[35] Ajlouni K., Khader Y., Batieha A., Jaddou H., El-Khateeb M. (2020). An alarmingly high and increasing prevalence of obesity in Jordan. Epidemiol. Health.

[36] Khabazkhoob M., Emamian M.H., Hashemi H., Shariati M., Fotouhi A. (2017). Prevalence of overweight and obesity in the middle-age population: A priority for the health system. Iran. J. Public Health.

[37] Løvsletten O., Jacobsen B.K., Grimsgaard S., Njølstad I., Wilsgaard T. (2020). Prevalence of general and abdominal obesity in 2015-2016 and 8-year longitudinal weight and waist circumference changes in adults and elderly: The Tromsø Study. BMJ Open.

[38] Oliveira A., Araújo J., Severo M., Correia D., Ramos E., Torres D., Lopes C. (2018). Prevalence of general and abdominal obesity in Portugal: Comprehensive results from the National Food, nutrition and physical activity survey 2015–2016. BMC Public Health.

[39] Wang L., Lee Y., Wu Y., Zhang X., Jin C., Huang Z., Wang Y., Wang Z., Kris-Etherton P., Wu S. (2020). A prospective study of waist circumference trajectories and incident cardiovascular disease in China: The Kailuan Cohort Study. Am. J. Clin. Nutr..

[40] Gurung M.S., Guwatudde D., Msaidié M., Houehanou C., Houinato D., Jorgensen J.M.A., Kagaruki G.B., Karki K.B., Labadarios D., Martins J.S. (2020). Diabetes Prevalence and Its Relationship with Education, Wealth, and BMI in Twenty-Nine Low-and Middle-Income Countries. Diabetes Care.

[41] Jeon J., Jung K.J., Jee S.H. (2019). Waist circumference trajectories and risk of type 2 diabetes mellitus in Korean population: The Korean genome and epidemiology study (KoGES). BMC Public Health.

[42] Zhao Q., Laukkanen J.A., Li Q., Li G. (2017). Body mass index is associated with type 2 diabetes mellitus in Chinese elderly. Clin. Interv. Aging.

[43] Khan S.S., Ning H., Wilkins J.T., Allen N., Carnethon M., Berry J.D., Sweis R.N., Lloyd-Jones D.M. (2018). Association of body mass index with lifetime risk of cardiovascular disease and compression of morbidity. JAMA Cardiol..

[44] Ganz M.L., Wintfeld N., Li Q., Alas V., Langer J., Hammer M. (2014). The association of body mass index with the risk of type 2 diabetes: A case–control study nested in an electronic health records system in the United States. Diabetol. Metab. Syndr..

[45] Bae J.C., Cho N.H., Kim J.H., Hur K.Y., Jin S.-M., Lee M.-K. (2020). Association of Body Mass Index with the Risk of Incident Type 2 Diabetes, Cardiovascular Disease, and All-Cause Mortality: A Community-Based Prospective Study. Endocrinol. Metab..

[46] de Koning L., Merchant A.T., Pogue J., Anand S.S. (2007). Waist circumference and waist-to-hip ratio as predictors of cardiovascular events: Meta-regression analysis of prospective studies. Eur. Heart J..

[47] Feller S., Boeing H., Pischon T. (2010). Body mass index, waist circumference, and the risk of type 2 diabetes mellitus: Implications for routine clinical practice. Dtsch. Ärzteblatt Int..

[48] World Health Organization Noncommunicable Diseases. https://www.who.int/news-room/fact-sheets/detail/noncommunicable-diseases.

[49] World Health Organization Diabetes. https://www.who.int/news-room/fact-sheets/detail/diabetes#.

[50] OECD (2020). Is Cardiovascular Disease Slowing Improvements in Life Expectancy?: OECD and the King’s Fund... Workshop Proceedings.

[51] Bhagavathula A., Shehab A., Ullah A., Rahmani J. (2020). The Burden of Cardiovascular Disease Risk Factors in the Middle East: A Systematic Review and Meta-Analysis Focusing on Primary Prevention. Curr. Vasc. Pharmacol..

[52] Al-Sahouri A., Merrell J., Snelgrove S. (2019). Barriers to good glycemic control levels and adherence to diabetes management plan in adults with Type-2 diabetes in Jordan: A literature review. Patient Prefer. Adherence.

[53] Jelwan Y.A., Asbeutah A.A.A., Welty F.K. (2020). Comprehensive Review of Cardiovascular Diseases, Diabetes, and Hypercholesterolemia in Lebanon. Cardiol. Rev..

[54] International Diabetes Federation (2017). IDF Diabetes Atlas.

[55] Nayak B.S., Sobrian A., Latiff K., Pope D., Rampersad A., Lourenço K., Samuel N. (2014). The association of age, gender, ethnicity, family history, obesity and hypertension with type 2 diabetes mellitus in Trinidad. Diabetes Metab. Syndr. Clin. Res. Rev..

[56] Silveira E.A., de Souza Rosa L.P., de Souza Cardoso C.K., Noll M. (2020). Type 2 diabetes mellitus in class II and III obesity: Prevalence, associated factors, and correlation between glycemic parameters and body mass index. Int. J. Environ. Res. Public Health.

[57] Martinell M., Pingel R., Hallqvist J., Dorkhan M., Groop L., Rosengren A., Storm P., Stålhammar J. (2017). Education, immigration and income as risk factors for hemoglobin A1c > 70 mmol/mol when diagnosed with type 2 diabetes or latent autoimmune diabetes in adult: A population-based cohort study. BMJ Open Diabetes Res. Care.

[58] Hwang J., Shon C. (2014). Relationship between socioeconomic status and type 2 diabetes: Results from Korea National Health and Nutrition Examination Survey (KNHANES) 2010–2012. BMJ Open.

[59] Toshihiro á., Saito K., Takikawa S., Takebe N., Onoda T., Satoh J. (2008). Psychosocial factors are independent risk factors for the development of Type 2 diabetes in Japanese workers with impaired fasting glucose and/or impaired glucose tolerance 1. Diabet. Med..

[60] Ruan Y., Guo Y., Zheng Y., Huang Z., Sun S., Kowal P., Shi Y., Wu F. (2018). Cardiovascular disease (CVD) and associated risk factors among older adults in six low-and middle-income countries: Results from SAGE Wave 1. BMC Public Health.

[61] Rosengren A., Smyth A., Rangarajan S., Ramasundarahettige C., Bangdiwala S.I., AlHabib K.F., Avezum A., Boström K.B., Chifamba J., Gulec S. (2019). Socioeconomic status and risk of cardiovascular disease in 20 low-income, middle-income, and high-income countries: The Prospective Urban Rural Epidemiologic (PURE) study. Lancet Glob. Health.

[62] Geldsetzer P., Manne-Goehler J., Theilmann M., Davies J.I., Awasthi A., Danaei G., Gaziano T.A., Vollmer S., Jaacks L.M., Bärnighausen T. (2018). Geographic and sociodemographic variation of cardiovascular disease risk in India: A cross-sectional study of 797,540 adults. PLoS Med..

[63] İzmirli M., Göktekin Ö., Bacaksız A., Uysal Ö., Kılıç Ü. (2015). The effect of the SIRT1 2827 A > G polymorphism, resveratrol, exercise, age and occupation in Turkish population with cardiovascular disease. Anatol. J. Cardiol..

[64] Zaitsu M., Kato S., Kim Y., Takeuchi T., Sato Y., Kobayashi Y., Kawachi I. (2019). Occupational class and risk of cardiovascular disease incidence in Japan: Nationwide, multicenter, hospital-based case-control study. J. Am. Heart Assoc..

[65] Wang S., Ma W., Yuan Z., Wang S.-m., Yi X., Jia H., Xue F. (2016). Association between obesity indices and type 2 diabetes mellitus among middle-aged and elderly people in Jinan, China: A cross-sectional study. BMJ Open.

[66] Feng R.-N., Zhao C., Wang C., Niu Y.-C., Li K., Guo F.-C., Li S.-T., Sun C.-H., Li Y. (2012). BMI is strongly associated with hypertension, and waist circumference is strongly associated with type 2 diabetes and dyslipidemia, in northern Chinese adults. J. Epidemiol..

[67] Li R., Shi L., Jia J., Li Y., Yang Q., Ruan Y., Chen R., Kan H. (2015). Differentiating the associations of waist circumference and body mass index with cardiovascular disease risk in a Chinese population. Asia Pac. J. Public Health.

[68] Borel A.-L., Coumes S., Reche F., Ruckly S., Pépin J.-L., Tamisier R., Wion N., Arvieux C. (2018). Waist, neck circumferences, waist-to-hip ratio: Which is the best cardiometabolic risk marker in women with severe obesity? The SOON cohort. PLoS ONE.

[69] Han T.S., Al-Gindan Y.Y., Govan L., Hankey C.R., Lean M.E.J. (2019). Associations of BMI, waist circumference, body fat, and skeletal muscle with type 2 diabetes in adults. Acta Diabetol..

[70] Nyamdorj R., Decoda Study Group (2008). BMI compared with central obesity indicators in relation to diabetes and hypertension in Asians. Obesity.

[71] Chen G.-C., Arthur R., Iyengar N.M., Kamensky V., Xue X., Wassertheil-Smoller S., Allison M.A., Shadyab A.H., Wild R.A., Sun Y. (2019). Association between regional body fat and cardiovascular disease risk among postmenopausal women with normal body mass index. Eur. Heart J..

[72] Janssen I., Katzmarzyk P.T., Ross R. (2004). Waist circumference and not body mass index explains obesity-related health risk. Am. J. Clin. Nutr..

[73] Regi M., Sharma N. (2016). Body Adiposity Index versus Body Mass Index and Other Anthropometric Traits as Correlates of Cardiovascular Disease. Int. J. Res. Sci. Innov. (IJRSI).

[74] Perea V., Jiménez A., Flores L., Ortega E., Coves M.J., Vidal J. (2013). Anthropometric indexes outperform bioelectrical impedance analysis-derived estimates of body composition in identification of metabolic abnormalities in morbid obesity. Surg. Obes. Relat. Dis..

[75] Park D., Lee J.-H., Han S. (2017). Underweight: Another risk factor for cardiovascular disease?: A cross-sectional 2013 Behavioral Risk Factor Surveillance System (BRFSS) study of 491,773 individuals in the USA. Medicine.

[76] Wu C.-Y., Chou Y.-C., Huang N., Chou Y.-J., Hu H.-Y., Li C.-P. (2014). Association of body mass index with all-cause and cardiovascular disease mortality in the elderly. PLoS ONE.

[77] Tennant I.A., Barnett A.T., Thompson D.S., Kips J., Boyne M.S., Chung E.E., Chung A.P., Osmond C., Hanson M.A., Gluckman P.D. (2014). Impaired cardiovascular structure and function in adult survivors of severe acute malnutrition. Hypertension.

[78] Grey K., Gonzales G.B., Abera M., Lelijveld N., Thompson D., Berhane M., Abdissa A., Girma T., Kerac M. (2021). Severe malnutrition or famine exposure in childhood and cardiometabolic non-communicable disease later in life: A systematic review. BMJ Glob. Health.

